# Senescence Phenomena and Metabolic Alteration in Mesenchymal Stromal Cells from a Mouse Model of Rett Syndrome

**DOI:** 10.3390/ijms20102508

**Published:** 2019-05-21

**Authors:** Tiziana Squillaro, Nicola Alessio, Stefania Capasso, Giovanni Di Bernardo, Mariarosa Anna Beatrice Melone, Gianfranco Peluso, Umberto Galderisi

**Affiliations:** 1 Department of Advanced Medical and Surgical Sciences, Center for Rare Diseases and Inter University Center for Research in Neurosciences, University of Campania “Luigi Vanvitelli”, via Sergio Pansini, 5, 80131 Naples, Italy; tiziana.squillaro@unicampania.it (T.S.); marina.melone@unicampania.it (M.A.B.M.); 2 Department of Experimental Medicine, Campania University “Luigi Vanvitelli”, via Santa Maria di Costantinopoli, 16, 80138 Naples, Italy; nicola.alessio@unicampania.it (N.A.); stefania.capasso@unicampania.it (S.C.); gianni.dibernardo@unicampania.it (G.D.B.); 3 Sbarro Institute for Cancer Research and Molecular Medicine, Department of Biology, BioLife Building (015-00)1900 North 12th Street, Temple University, Philadelphia, PA 19122-6078, USA; 4 Research Institute on Terrestrial Ecosystems, National Research Council, via Pietro Castellino, 111, 80131 Naples, Italy; gianfranco.peluso@cnr.it

**Keywords:** Rett syndrome, mesenchymal stromal cells, senescence, DNA repair, metabolism, ATP levels, mitochondrial ATP, autophagy, proteasome

## Abstract

Chromatin modifiers play a crucial role in maintaining cell identity through modulation of gene expression patterns. Their deregulation can have profound effects on cell fate and functions. Among epigenetic regulators, the MECP2 protein is particularly attractive. Mutations in the *Mecp2* gene are responsible for more than 90% of cases of Rett syndrome (RTT), a progressive neurodevelopmental disorder. As a chromatin modulator, MECP2 can have a key role in the government of stem cell biology. Previously, we showed that deregulated MECP2 expression triggers senescence in mesenchymal stromal cells (MSCs) from (RTT) patients. Over the last few decades, it has emerged that senescent cells show alterations in the metabolic state. Metabolic changes related to stem cell senescence are particularly detrimental, since they contribute to the exhaustion of stem cell compartments, which in turn determine the falling in tissue renewal and functionality. Herein, we dissect the role of impaired MECP2 function in triggering senescence along with other senescence-related aspects, such as metabolism, in MSCs from a mouse model of RTT. We found that MECP2 deficiencies lead to senescence and impaired mitochondrial energy production. Our results support the idea that an alteration in mitochondria metabolic functions could play an important role in the pathogenesis of RTT.

## 1. Introduction

Rett syndrome (RTT; OMIM #312750) is a complex and devastating neurodevelopmental disease caused in more than 90% of RTT cases by loss-of-function mutations in the X-linked *Mecp2* gene encoding methyl-CpG binding protein 2 (MECP2) [1]. The MECP2 protein was initially identified as a transcriptional repressor given its capacity to bind methylated DNA and mediate gene silencing by triggering modification of chromatin architecture [2,3]. Later, it was described as a multifunctional modulator of gene expression with activating or repressing functions depending on the molecular context [4]. The MECP2 protein is ubiquitously expressed, but the highest expression levels are found in the brain [5,6]. It has been widely reported that *Mecp2* mutations can impair the functionality of many genes both in nervous and other tissues (such as muscle and bone) [3,7,8,9]. However, even if the knowledge of MECP2 target genes is not yet complete, the role of this gene in the maintenance of chromatin architecture has been clearly identified. For this reason, some researchers identify RTT, which is caused by mutations in the *Mecp2* gene, as ‘a paradigmatic example of a chromatin disorder’ [10]. As a chromatin modulator, MECP2 can have a key role in the government of stem cell biology. Indeed, several aspects of stem cell life are regulated by epigenetic modifications that, for example, may repress the expression of genes involved in lineage specification and promoting the induction of those involved in stemness maintenance [11]. Moreover, epigenetic variations may also be involved in the impairment of stem cell physiological functions [11,12]. Stem cells undergo changes in chromatin dynamics and gene expression profiling when they senesce. This process, due to derangement of chromatin modifiers, can be induced by several exogenous and endogenous stresses. Accordingly, *Mecp2* mutations can also alter the physiological activity of stem cells [3,7]. Understanding the MECP2 role in the regulation of stem cell biology can have a profound impact on the life of an individual. In a previous study, we showed that mesenchymal stromal cells (MSCs) obtained from the bone marrow of RTT patients are prone to senescence [8]. These results were validated in an in vitro model of MECP2 partial silencing [3]. Recently, we demonstrated that mouse neural stem cells with impaired MECP2 function are affected by premature senescence [7,9].

Over the last few decades, it has emerged that senescent cells show alterations in the metabolic state. In particular, the proper functioning of stem cell metabolism is of great importance, since it is involved in regulating the balance between quiescence, proliferation, and differentiation [13,14]. Metabolic changes related to stem cell senescence could contribute to exhaustion of stem cell compartments, which in turn determine the fall in tissue renewal and functionality [15]. It has also been demonstrated that senescence occurs as a result of the accumulation of detrimental changes over time and that this may be due to improper function of the DNA repair system activation, autophagy process, and/or proteasome activity [16,17,18].

In the present paper, we aim to further clarify the role of impaired MECP2 function in triggering senescence. To this purpose, we decided to dissect the senescence process along with other senescence-related cellular aspects, such as the DNA repair system, metabolism, autophagy process, and proteasome activity in MSCs from a mouse model of RTT.

## 2. Results

We decided to use heterozygote female mice of the B6.129P2(C)-Mecp2^tm1.1Bird^/J strain to evaluate the effects of partial MECP2 loss of function, since this heterozygosity condition may occur in girls with RTT. Indeed, in a previous in vitro study, we demonstrated that even the partial silencing of the *Mecp2* gene may impair stem cell biology [3].

In the present study, we isolated MSCs from the bone marrow of *Mecp2^+/−^* and wild type (WT) mice and analyzed their biological properties. We chose MSCs given their important role in supporting hematopoiesis and contributing to homeostasis of several organs and tissues. Moreover, MSCs contain a subpopulation of stem cells able to differentiate in osteocytes, adipocytes, and chondrocytes [19,20,21,22,23]. In addition, as progenitors of osteocytes, studying MSC biology could be of interest, since it has been reported that RTT patients develop several skeletal abnormalities, such as low bone density, high frequency of fractures, and scoliosis [24].

### 2.1. MSCs from Mecp2^+/−^ Mice Showed a Lower Degree of Proliferation and Apoptosis and Are Prone to Senescence

**Figure 1:**
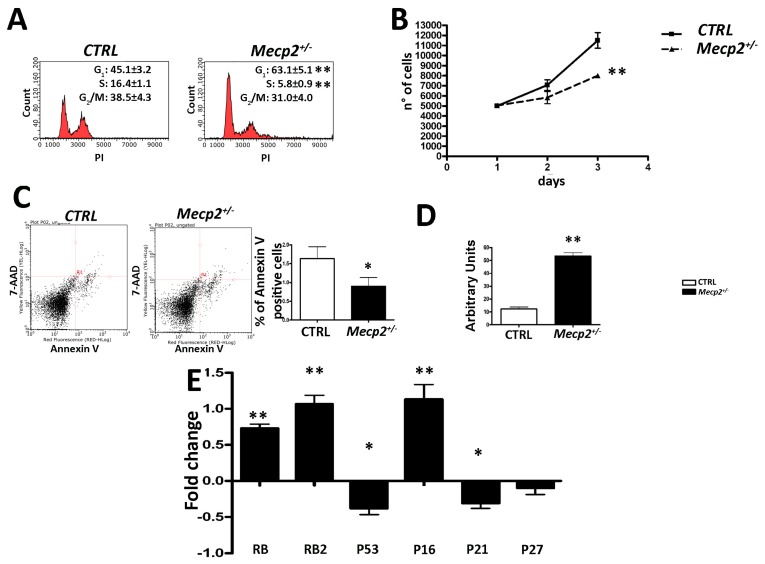
Cell growth, apoptosis, and senescence evaluation. (**A**) The picture shows the cell cycle profiles of the *Mecp2^+/−^* mesenchymal stromal (MSCs) and control (CTRL) cells (±SD, *n* = 5 biological replicates, ** *p* < 0.01). (**B**) Cell proliferation evaluation by the Quick Cell Proliferation Assay Kit II. At 1, 2, and 3 days post plating, the cells were collected and counted (±SD, *n* = 5 biological replicates, ** *p* < 0.01). (**C**) Apoptosis detection by the fluorescein conjugated Annexin V assay. Apoptotic cells were identified with a fluorescence microscope. The figure shows a representative apoptosis profile of the *Mecp2^+/−^* and CTRL MSCs. The graph shows mean expression values in the *Mecp2^+/−^* and CTRL MSCs (±SD, *n* = 5 biological replicates, * *p* < 0.05). (**D**) Senescence analysis by the 4-methylumbelliferyl-β-d -galactopyranoside (MUG) quantitative fluorescent assay. 4-MUG is a β-galactosidase substrate that emits fluorescence only when it is cleaved by the enzyme to generate fluorophore 4-methylumbelliferone. The fluorophore production was monitored at an emission/excitation wavelength of 365/460 nm. The data are expressed as arbitrary units (±SD, *n* = 5 biological replicates, ** *p* < 0.01). (**E**) RT-qPCR analysis. The picture shows the mRNA expression levels of *Rb1*, *Rb2/P130*, *P53*, *P21*, and *P27* in the *Mecp2^+/−^* and CTRL MSCs. *Hprt* mRNA was used as an internal control. Data are expressed as fold change using delta-delta CT method (±SD, *n* = 5 biological replicates, * *p* < 0.05, ** *p* < 0.01).

MSCs from the *Mecp2^+/−^* and WT mice were analyzed by flow cytometry. As shown in Figure 1A, we noticed a significant reduction of S-phase cells, along with an increase of G_1_ cells in the *Mecp2^+/−^* MSCs with respect to the control (CTRL) cells. The decrease of cells in S-phase was in agreement with the decrement of cell proliferation evaluated by counting cells at different time intervals (Figure 1B).

The annexin assay showed a significant reduction in apoptotic cell percentage in the *Mecp2^+/−^* samples when compared to the CTRL ones, although the apoptosis levels were very low in both the *Mecp2^+/−^* and CTRL MSCs (Figure 1C). Of interest, the β-galactosidase assay detected a higher percentage of senescent cells in MSCs from the *Mecp2^+/−^* mice with respect to the CTRL cells (Figure 1D). To extend this finding, we evaluated changes in the expression levels of the Retinoblastoma family genes (i.e., *Rb1* and *Rb2/P130*) and the tumor suppressor *P53* gene, which are involved in the regulation of cell cycle, differentiation, apoptosis, and senescence. Moreover, we analyzed the expression of some cyclin kinase inhibitors (CKIs), such as *P16^INK4A^*, *P21^CIP1^*, and *P27^KIP1^*, whose pathways overlap with those of the *RB* family and *P53* [25,26,27,28,29,30]. In the *Mecp2^+/−^* MSCs, we observed a significant increase of *Rb*, *Rb2/P130*, and *P16^INK4A^* mRNA levels when compared to the CTRL cells. Conversely, *P53* and *P21^CIP1^* mRNAs were significantly down-regulated in MSCs from the *Mecp2^+/−^* mice with respect to the CTRL cells. No significant differences were observed for the *P27* expression levels (Figure 1E).

As a whole, these data are in line with our previous studies on MSCs from RTT patients and an in vitro model of *Mecp2* gene silencing [3,7,8].

### 2.2. Impaired DNA Repair System in MSCs from Mecp2^+/−^ Mice

Following the generation of DNA damage, cells activate a signaling cascade known as DNA damage response (DDR). DDR activation leads to transient cell cycle arrest until the DNA damage has been completely repaired. When the DNA damage is not repaired, cell fate is directed toward apoptosis or senescence [31,32]. Indeed, following different senescence-inducing genotoxic stimuli, such as physical and chemical agents, the DDR pathway is active [28,32,33].

In this context, the senescence signs observed in the *Mecp2*^+/−^ MSCs prompted us to evaluate the DDR activation following genotoxic stress treatment (i.e., peroxide hydrogen-H_2_O_2_, doxorubicin, and UV irradiation) in our samples. This experiment was performed to investigate the DNA repair capacity of the *Mecp2^+/−^* MSCs by determining the number of γ-H2AX positive DNA foci in both basal condition and following genotoxic agent treatments. Following each genotoxic treatment, we also analyzed the levels of necrosis, apoptosis, and senescence in the *Mecp2^+/−^* and CTRL cells. Soon after DNA damage, cell cycle arrest is associated with activation of ataxia-telangiectasia mutated kinase (ATM) by autophosphorylation on Ser1981. This protein recognizes and binds DNA-damage foci contributing to the recruitment of the DNA repair system enzymes [34,35]. An important downstream target of ATM is the H2AX protein. Indeed, after ATM activation, the histone H2AX is phosphorylated and involved in the recruitment or retention of DNA repair system proteins. The phosphorylated H2AX (γ-H2AX) foci indicate damaged DNA that needs to be repaired, and their permanence for several hours or days following induction of DNA damage indicates the presence of misrepaired or unrepaired DNA foci [21,36,37].

**Figure 2:**
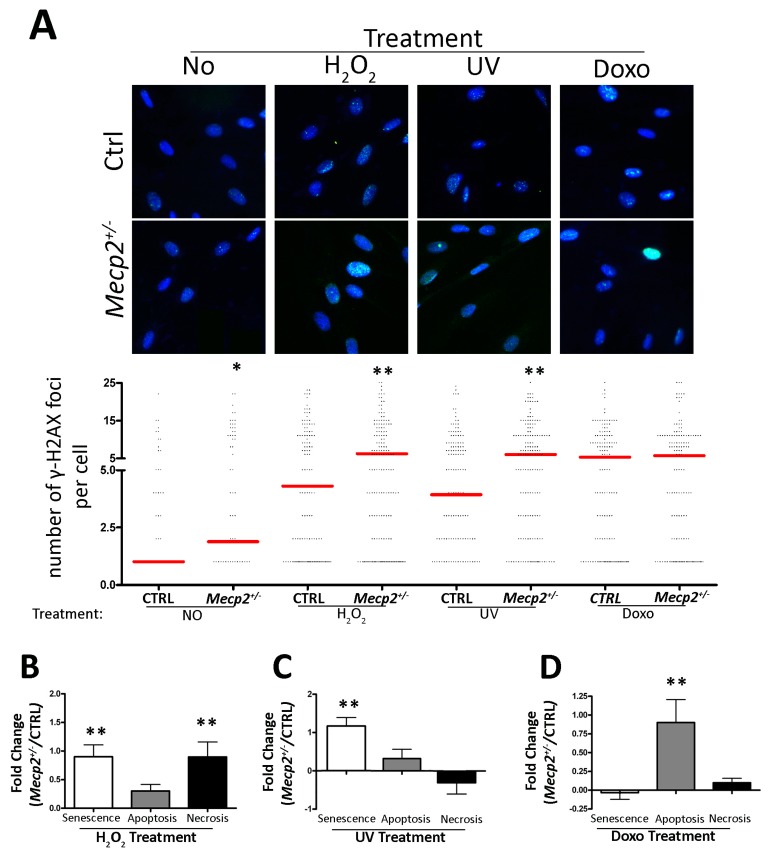
DNA damage, senescence, apoptosis, and necrosis. (**A**) Representative microscopic fields showing the merging of the *Mecp2^+/−^* and CTRL MSCs stained with anti-γ-H2AX (green) and 4′,6-diamidino-2-phenylindole (DAPI) (blue). The column scatter plot indicates the degree of H2AX phosphorylation that was determined by counting the number of γ-H2AX immunofluorescent foci per cell. The foci number was determined for 200 cells. Each dot represents a single cell. The horizontal bars indicate the mean value for each category (*Mecp2^+/−^* and CTRL MSCs; *n* = 5 biological replicates; * *p* < 0.05; ** *p* < 0.01). (**B**–**D**) The DNA damage levels 48 h following genotoxic treatments with hydrogen peroxide (H_2_O_2_), UV irradiation, and doxorubicin, respectively. For every condition, the degree of DNA damage was determined by counting γ-H2AX foci. Changes in the level of senescence, apoptosis, and necrosis in the *Mecp2^+/−^* MSCs were compared with the CTRL cells 48 h post DNA damage treatment. Senescence was analyzed by the 4-methylumbelliferyl-β-d galactopyranoside (MUG) quantitative fluorescent assay. 4-MUG is a β-galactosidase substrate that emits fluorescence only when it is cleaved by the enzyme to generate fluorophore 4-methylumbelliferone. The fluorophore production was monitored at an emission/excitation wavelength of 365/460 nm. Apoptosis was detected with the fluorescein conjugated Annexin V Kit and apoptotic cells were identified with a fluorescence microscope. Necrotic cells were detected by using Trypan blue staining. Non-viable cells were identified with an inverted microscope. The data are expressed in fold change (±SD, *n* = 5 biological replicates; * *p* < 0.05, ** *p* < 0.01).

We observed an increase in the number of γ-H2AX positive DNA foci in the *Mecp2^+/−^* MSCs in basal condition with respect to the CTRL cells (Figure 2A). These data led us to hypothesize that partial MECP2 deficiency may render MSCs more susceptible to DNA damage. Indeed, following H_2_O_2_ treatment, the MSCs from mutated mice showed a significant increase in the number of γ-H2AX positive DNA foci per cell when compared with CTRL samples. These foci persisted even 48 h following stress induction, suggesting the presence of unrepaired DNA foci (Figure 2A). These data were associated with a higher degree of necrosis and senescence while the apoptosis was reduced (Figure 2B). Treatment with UV revealed the same outcomes regarding the increase in the number of γ-H2AX positive DNA foci in the *Mecp2^+/−^* samples (Figure 2A). In this case, the *Mecp2^+/−^* MSCs entered senescence more often than the CTRL cells while apoptosis and necrosis were unchanged (Figure 2C). Treatment with doxorubicin did not reveal a significant difference in the number of γ-H2AX positive DNA foci in the *Mecp2^+/−^* MSCs when compared with the CTRL cells (Figure 2A). Moreover, the MSCs from the *Mecp2^+/−^* mice showed a higher percentage of apoptotic cells while necrosis and senescence remained unchanged (Figure 2D).

### 2.3. Mecp2^+/−^ MSCs Exhibited Metabolic Flexibility but Mitochondrial Energy Production Impairment

Metabolic flexibility is the ability of a cell to respond or adapt energy needs to fuel availability [38]. As a result, a healthy organism can switch among the principal nutrients (carbohydrates, amino acids, and lipids) according to physiological needs and environmental cues [15,39]. Nevertheless, metabolic flexibility is lost both in aging and age-related diseases [40,41]. For this reason, we decided to assess whether metabolic inflexibility was present in *Mecp2^+/−^* MSCs, since these cells are prone to senescence.

We cultivated the *Mecp2^+/−^* and CTRL MSCs in minimal media supplemented with either glucose, palmitate, or glutamine. Then, we determined ATP levels, lactate production, oxygen employed for mitochondrial ATP production, maximal respiration, and basal oxygen consumption rate in both the presence and absence of specific metabolic inhibitors, which are etomoxir, 2-deoxy-d-glucose (2-DG) and bis-2-(5-phenylacetamido-1,3,4-thiadiazol-2-yl)ethyl sulfide (BPTES), respectively. The effective concentrations of drugs were previously determined by a Trypan blue exclusion assay in order to avoid a potential cytotoxic effect of metabolic inhibitors (data not shown).

**Figure 3:**
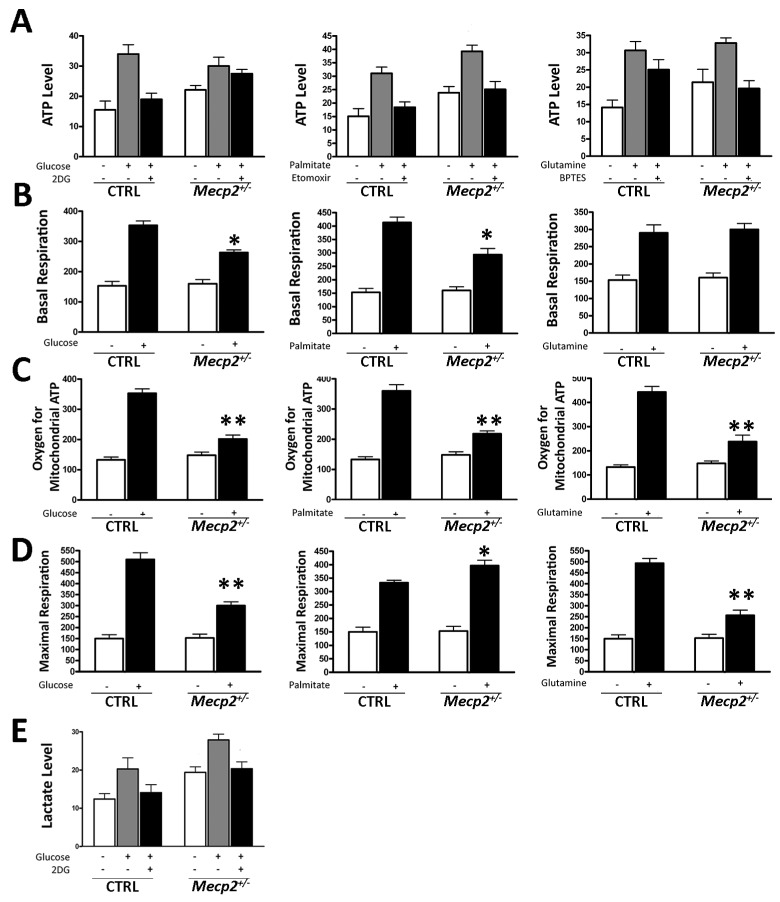
Evaluation of metabolic activity. (**A**) Intracellular ATP level evaluation by the ATP colorimetric/fluorometric assay. The cells were incubated in a medium containing glucose (left graph), palmitate (middle graph), or glutamine (right graph). The ATP level was measured in the absence of an energy substrate (white bars), in the presence of a specific substrate (gray bars), and in the presence of a substrate and its inhibitor (black bar). The data are expressed as ATP concentration (nM) (±SD, *n* = 5 biological replicates). (**B**) Basal oxygen consumption rate. We used the MitoXpress® Xtra assay for measurement of extracellular oxygen consumption rates (OCR). Cells were incubated in a medium containing glucose (left graph), palmitate (middle graph), or glutamine (right graph). For every condition, the asterisk (*) denotes significant differences (*p* < 0.05) between OCR in the *Mecp2^+/−^* versus CTRL MSCs. Data are expressed in arbitrary units (±SD, *n* = 5 biological replicates). (**C**) Oxygen for mitochondrial ATP production. OCR was measured following sequential addition of oligomycin that is an inhibitor of the F0 part of H+/ATP synthase in mitochondria. The cells were incubated in a medium containing glucose (left graph), palmitate (middle graph), or glutamine (right graph). For every condition, the asterisk denotes significant differences (** *p* < 0.01) between OCR in *Mecp2^+/−^* versus CTRL MSCs. Data are expressed in arbitrary units (±SD, *n* = 5 biological replicates). (**D**) Maximal respiration rate. OCR was measured following sequential addition of FCCP, that is, a protonophore and uncoupler of oxidative phosphorylation in mitochondria. The cells were incubated in a medium containing glucose (left graph), palmitate (middle graph), or glutamine (right graph). For every condition, the asterisk denotes significant differences (* *p* < 0.05; ** *p* < 0.01) between maximal OCR in the *Mecp2^+/−^* versus CTRL MSCs. Data are expressed in arbitrary units (±SD, *n* = 5 biological replicates). (**E**) Lactate level evaluation by the Lactate Assay Kit. The cells were incubated in a medium containing glucose with and without 2D-glucose, an inhibitor of glycolysis flux. The data are expressed as lactate concentration (nM) (±SD, *n* = 5 biological replicates).

The *Mecp2^+/−^* MSCs showed metabolic flexibility, which was also observed in the healthy CTRL cells (Figure 3A). Indeed, when the *Mecp2^+/−^* MSCs were cultured in a medium containing only glucose, palmitate, or glutamine, their ATP significantly increased with respect to a medium devoid of the specific nutrient (Figure 3A).

Then, we measured the intracellular oxygen to determine the carbohydrate, lipid, and glutamine contribution to oxygen consumption levels in the *Mecp2^+/−^* and CTRL MSCs. We first determined basal respiration, that is, the oxygen consumption used to meet cellular ATP demand. In media with glucose or palmitate, the basal respiration decreased in the *Mecp2^+/−^* cells when compared to the CTRL cells (Figure 3B). Conversely, in media containing glutamine as the nutrient, the basal oxygen consumption of the *Mecp2^+/−^* MSCs was not reduced compared to the CTRL cells (Figure 3B). Subsequently, we determined the oxygen employed for mitochondrial ATP production and maximal respiration. The ability to produce mitochondrial ATP in the presence of glucose, palmitate, or glutamine was significantly impaired in cells from the *Mecp2^+/−^* mice with respect to the CTRL cells (Figure 3C).

The measurement of maximal respiratory capacity (oxygen consumption after the injection of an oxidative phosphorylation uncoupler, such as carbonyl cyanide-4-(trifluoromethoxy) phenylhydrazone -FCCP), reveals how cells respond to an increase in ATP demand. The cell’s ability to react to sudden changes in the ATP requirement mainly depends on the energetic capacity of mitochondria. Only in media containing palmitate as the energy fuel did the *Mecp2^+/−^* MSCs show the capacity to cope with an increase in ATP demand when compared with the healthy CTRL cells (Figure 3D). As shown in Figure 3D, this ability was lost in media containing glucose or glutamine.

Finally, we also evaluated the ability of *Mecp2^+/−^* cells to produce lactate. Proliferating healthy cells depends on the first part of the glycolysis process to generate ATP and to reduce the NAD^+^/NADH ratio in the cytoplasm. Most of the resulting pyruvate is converted into lactate by the lactate dehydrogenase (LDH) enzyme, which also converts NADH in NAD+. Then, the lactate is secreted from the cells, and the produced NAD^+^ allows the glycolysis process to begin again [42].

As shown in Figure 3E, the *Mecp2^+/−^* MSCs did not lose the ability to produce lactate with respect to the CTRL cells, suggesting that they can undergo aerobic glycolysis for energy production.

### 2.4. Autophagy Was Not Impaired in Mecp2^+/−^ MSCs

**Figure 4:**
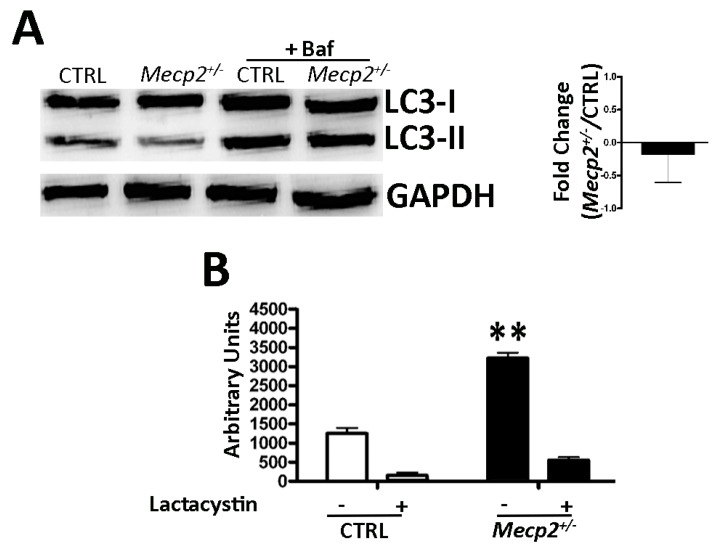
Autophagy and proteasome activity assays. (**A**) Autophagic flux evaluation. The picture shows western blot detection of LC3-I and LC3-II in the *Mecp2^+/−^* and CTRL MSCs. *Mecp2^+/−^* and CTRL MSCs were incubated with 100 nM bafilomycin A1 (inhibitor of lysosomal degradation) or phosphate-buffered saline (PBS) to detect autophagic flux. We used Gel Doc 2000 Gel Documentation Systems (Bio-Rad, Hercules, CA, USA) to measure LC3-I and II band intensities that were normalized with GAPDH as internal control. We determined autophagic flux (AF) for LC3 II as follows: Mecp2^+/−^ MSC AF = (Mecp2^+/−^ MSCs + Bafilomycin A1) − (Mecp2^+/−^ MSCs + PBS); CTRL MSC AF = (CTRL MSCs + Bafilomycin A1) − (CTRL MSCs + PBS). Change in autophagic flux (ΔAF) between Mecp2^+/−^ and CTRL MSCs was calculated as follows: ΔAF = Mecp2^+/−^ MSC AF − CTRL MSC AF. The graph shows AF changes in the Mecp2^+/−^ MSCs compared to CTRL cells. The data are expressed in change folds (±SD, *n* = 5). (**B**) Proteasome activity. Mecp2^+/−^ and CTRL cells were harvested for a fluorometric assay determination of proteasome activity. Mecp2^+/−^ and CTRL cultures were incubated with 25 μM lactacystin (a specific inhibitor of proteasomes) or PBS. The graph shows proteasome activity in the Mecp2^+/−^ and CTRL cells both in the presence and absence of lactacystin. Data are expressed in arbitrary units (±SD, *n* = 5; ** *p* < 0.01) The asterisk denotes significant differences between Mecp2^+/−^ samples versus CTRL ones.

To determine the effect of the *Mecp2* mutation on the autophagic flux, we analyzed by western blot the levels of LC3-I and LC3-II, two isoforms of the microtubule-associated protein 1 light chain 3 (LC3), which is a valid marker of autophagosome. Following the autophagy induction, the autophagic flux can be tracked by measuring the conversion of LC3-I proteins to LC3-II. At a given point, the LC3 levels do not indicate autophagic flux. Thus, it is important to measure the amount of LC3-II in both the presence and absence of lysosomal protease inhibitors to evaluate changes in the autophagic flux [43]. We determined the LC3-I and LC3-II levels, both in the absence and presence of bafilomycin A1, an inhibitor of lysosomal degradation. In the *Mecp2^+/−^* MSCs, we observed no differences in the autophagic flux when compared with the CTRL cells (Figure 4A).

### 2.5. Mecp2^+/−^ Mice Showed an Increase in Proteasome Activity

In the eukaryotic cell, the majority of proteins are degraded by the activity of the ubiquitin-proteasome pathway. It catalyzes the selective degradation of regulatory proteins (short half-life proteins) and of those damaged or with abnormal conformation [44]. To evaluate the intracellular ubiquitin-proteasome activity, we measured the activity of 20S proteasome, the catalytic unit of the proteasome complex, by a specific assay. The detection of proteasome activity was performed both in the presence and absence of lactacystin, a selective and irreversible proteasome inhibitor [45]. If in the presence of the protease inhibitor, the 20S proteasome activity is not reduced. This indicates an impairment of proteasome functionality. The *Mecp2^+/−^* MSCs showed a significant increase in proteasome activity with respect to the CTRL cells. Moreover, in the presence of lactacystin, the proteasome activity adequately decreased, as observed in the CTRL cells (Figure 4B).

## 3. Discussion

Chromatin modifiers play a key role in maintaining cell identity through modulation of gene expression patterns. Their deregulation can have profound effects on cell fate and function. Indeed, many chromatin remodelers are often found deregulated in aging and several human diseases, including cancer [46,47]. Among epigenetic regulators of the genome, the MECP2 protein is particularly attractive. Indeed, mutations in the *Mecp2* gene are responsible for more than 90% of RTT cases, a progressive and complex neurodevelopmental disorder that occurs almost exclusively in females [1,10]. In previous findings, we showed that deregulated MECP2 expression triggers a senescence process in MSCs from RTT patients and in an in vitro model of partial gene silencing [3,7,8].

Here, we further dissected the role of MECP2 in senescence regulation along with other senescence-related cellular aspects, such as the DNA repair system, metabolism, autophagy process, and proteasome activity, in MSCs from a mouse model of RTT. Our results showed that the impaired expression of MECP2 is related to the cell cycle arrest in G_1_, reduction in apoptosis, and an increase in senescence (Figure 1). Cell cycle arrest and senescence process seems to be related to RB-P16 or RB2/P130-P16 pathway activation, as shown by mRNA expression analysis (Figure 1E). These data are consistent with findings demonstrating that P16-mediated senescence acts through the retinoblastoma pathway to inhibit the action of the cyclin-kinases determining cell cycle arrest in the G_1_ phase [25,48,49,50]. Interestingly, senescence observed in the *Mecp2^+/−^* cells was connected with an accumulation of unrepaired or misrepaired DNA foci (Figure 2A). Indeed, the *Mecp2^+/−^* MSCs were not able to cope with genotoxic agents as well as the CTRL cells. Following treatment with H_2_O_2_ and UV, the MSCs with impaired MECP2 expression showed an increase in γ-H2AX^+^ DNA foci and were more prone to senescence with respect to the CTRL cells (Figure 2B,C). Conversely, in doxorubicin-treated *Mecp2^+/−^* MSCs, we observed the onset of cell death when compared with the CTRL cells (Figure 2D). In this latter case, the increased cell susceptibility to doxorubicin treatment could be due to the characteristics of the stressor agent rather than a total failure to counteract toxic events. In fact, it has to be taken into account that senescent cells lose their proliferation and growth capacity but are still alive while apoptotic cells are programmed to die. The susceptibility of MSCs with an impaired MECP2 function to accumulate unrepaired DNA damage is in compliance with literature data showing that DNA repair system performance is critically influenced by alterations in chromatin remodeling [51,52,53,54].

An extensive analysis of metabolic needs in *Mecp2^+/−^* MSCs was of interest given the involvement of these phenomena in triggering and/or sustaining the senescence process. It has to be taken into account that MSC cultures contain stem cells with different multipotent properties, committed progenitors, and differentiated cells [22]. Metabolic requirements strictly depend on the energetic demands of different cell states (proliferation, differentiation, quiescence, senescence). To satisfy the anabolic requirements of proliferating and self-renewing stem cells, a compromise between catabolic ATP generation, reducing cofactors and substrates (i.e., anaerobic glycolysis and pentose phosphate pathway), is needed. Meanwhile, in progenitors and differentiated cells, ATP production through the tricarboxylic acid (TCA) cycle is required to accomplish the large amounts of energy to support tissue homeostasis and specialized function [15,55]. Thus, healthy MSCs are able to produce ATP by both the TCA cycle and anaerobic glycolysis.

Our data showed that *Mecp2^+/−^* cells possess a metabolic flexibility. Indeed, cells with impaired MECP2 function had the ability to switch among alternative nutrients (carbohydrates, lipids, and amino acids) to ATP production according to environmental cues as they happen in physiological conditions (Figure 3A). Similarly, *Mecp2^+/−^* cells did not lose the ability to produce lactate, suggesting that they showed the capacity to undergo aerobic glycolysis for energy production (Figure 3E).

Concerning intracellular oxygen consumption, in the *Mecp2^+/−^* cells, we noted a decrease of basal respiration in the presence of glucose or palmitate as well as a reduced capacity to produce mitochondrial ATP (Figure 3B,C). These data may indicate that efficiency in ATP production is impaired in *Mecp2^+/−^* cells. Indeed, the *Mecp2^+/−^* MSCs showed a decrease in maximal respiratory capacity in media containing either glucose or glutamine (Figure 3D). An analysis of maximal respiratory capacity allowed us to evaluate how cells can respond to an increase in ATP requirement. This ability, which mainly depends on the energy-generating capacity of mitochondria, is partially lost in *Mecp2^+/−^* cells (Figure 3D). Of great interest, the *Mecp2^+/−^* cells can cope with a higher energy demand only with palmitate (Figure 3D). This result is in good agreement with findings showing that RTT patients may have symptom relief by treatment with a ketogenic diet, which is based mainly on lipid supplementation [56,57].

Globally, our data are in agreement with several reports showing an altered mitochondrial structure along with deficiencies in activity of mitochondrial enzymes in cells and tissues from RTT patients and in model systems [58]. Moreover, our group has recently demonstrated the presence of mitochondrial dysfunction in cardiac tissue from an in vivo RTT model [59]. Accordingly, it has been reported that RTT shares a lot of similarities with mitochondrial diseases, such as elevated levels of lactate and pyruvate in cerebrospinal fluid and blood as well as increased oxidative damage and impairment of electron transport chain function [58,60,61,62]. In a fascinating study, Park et al. reported that some of the deleterious symptoms of RTT may be related to energy deficits due to abnormalities in substrates’ utilization of the TCA cycle. The administration of a diet providing alternative intermediate substrates in Mecp2-null mice restored metabolic homeostasis and improved energy availability that would be involved in neurological and neuromuscular deficiencies [63].

In our experimental model, senescence induced by impaired MECP2 function was not related to autophagy dysfunction as can occur in some contexts (Figure 4A). The degradation of cytoplasmic components by autophagy enables cells to survive external and internal stresses. In this scenario, autophagy protects cells from stress-induced damage—if autophagy is impaired, it may trigger a senescence process. On the other hand, it has also been reported that autophagy may promote senescence depending on stressors and cellular context [16,17]. We may hypothesize that cells with partial function of MECP2 were still able to remove damaged components by activating autophagy flux. In line with this finding, we observed an increase of proteasome activity in the *Mecp2^+/−^* MSCs with respect to the CTRL cells (Figure 4B). It is widely reported that during aging, a large number of misfolded, oxidized, and cross-linked proteins accumulate in cells, impairing cell function and tissue homeostasis [18,64,65]. We hypothesized that *Mecp2^+/−^* cells upregulate the intracellular protease complex activity to cope with the over-accumulation of non-functioning proteins. Over time, this effort could lead to the decline of cell proteasomal machinery, which occurs in aging.

The proper functioning of stem cell compartments is of great importance, since it is involved in regulating the balance between quiescence, proliferation, and differentiation [13,14]. When senescence occurs in these cellular compartments, it is particularly detrimental for an organism, since it could contribute to stem cell exhaustion, which in turn determines the falling in tissue renewal and functionality [15]. Our study indicated that senescence of MSCs may be associated with impaired function of MECP2.

To our knowledge, we were the first research group to identify an association between RTT syndrome and senescence [3,7,8,9]. In agreement with our data, Ohashi et al. recently demonstrated that RTT patient-derived neurons showed signs of stress, such as H2AX deposition, induction of aging-related genes, and senescence phenomena [66]. Literature data reported that RTT patients showed normal post-natal growth and development followed by a development stop, stereotyped hand movements, seizure, walking problems, intellectual disability, and loss of the gained language skills at the end of second year. Decades later, survivors show parkinsonian phenotype [67,68,69,70]. Currently, there are no studies showing a premature aging in the RTT patients.

Future analyses are needed to better investigate our hypothesis as well as evaluate the contribution of mitochondrial energy production impairment in triggering and/or sustaining the senescence observed in RTT MSCs. As a whole, our future research may elucidate whether the loss of MECP2 functionality is involved in triggering premature aging.

## 4. Materials and Methods

### 4.1. Animals

Heterozygous B6.129P2(C)-Mecp2^tm1Bird^/J (*Mecp2^+/−^*) female mice (Jackson Laboratories, Bar Harbor, ME, USA) (JAX stock: 003890) were bred with C57BL/6 wild-type male mice (Jackson Laboratories) (JAX stock: 000664), as indicated on the website of Jackson Laboratories (https://www.jax.org/) [71,72]. Wild-type (*Mecp2^+/+^*) female littermates were used as the control. As expected, heterozygous females showed RTT onset at around 6 months of age. Mice were handled in compliance with protocols that were approved by the Animal Care and Use Committee of the University of Campania “Luigi Vanvitelli”, Naples, Italy. Ethics committee approval nr.317/2016 PR, Italian Ministry of Health, Italy (29 March 2016). The *Mecp2^+/−^* and wild-type (WT) mice were euthanized via cervical dislocation under anesthesia.

### 4.2. Genotyping

Genomic DNA was extracted from two centimeters of tail tips using the DNeasy Blood & Tissue kit (Qiagen Italia, Milan, Italy), following the manufacturer’s instructions. Then, we determined the genotype of the mice by polymerase chain reaction (PCR), as reported on the website of Jackson Laboratories (https://www.jax.org/).

### 4.3. Mouse MSC Cultures

MSCs were harvested from the bone marrow of femurs and tibias of the *Mecp2^+/−^* and WT mice euthanized as above reported. We inserted a 21-gauge needle into the shaft of the bone and flushed it with Minimal Essential Media with alpha modifications (α-MEM). Then, we collected and plated cells from each mouse onto two 100-mm dishes with α-MEM containing 10% fetal bovine serum (FBS) and basic fibroblast growth factor (bFGF). After 24 to 48 h, non-adherent cells were discarded while adherent ones were washed twice with phosphate-buffered saline (PBS). Cells were incubated for 7 to 10 days in proliferating medium to the confluence and then propagated to conduct experiments at passages 3 or 4. Cells were grown in monolayers and maintained at 37 °C under 5% CO_2_. The cell culture reagents were purchased from EuroClone S.p.A, Pero, Italy.

### 4.4. Cell Cycle Analysis

The *Mecp2^+/−^* and CTRL MSCs were fixed in 70% ethanol, washed in PBS, and then stained with a hypotonic propidium iodide solution. The samples were read with a Guava EasyCyte flow cytometer (Merck Millipore, Milano, Italy) and analyzed by the EasyCyte software, following the manufacturer’s procedure.

### 4.5. Proliferation Analysis

The cell proliferation rate was evaluated with the Quick Cell Proliferation Assay Kit II (Biovision, Milpitas, CA, USA). The colorimetric assay allowed us to determine the number of living cells by measuring the cleavage of the tetrazolium salt to formazan by cellular mitochondrial dehydrogenase. The formazan dye produced by viable cells was then quantified by measuring its absorbance at 440 nm in a 96-well spectrophotometer.

### 4.6. Senescence Detection

The senescence rate was evaluated with a quantitative senescence-associated β-galactosidase assay. Briefly, 4-methylumbelliferyl-β-d-galactopyranoside (4-MUG) is a β-galactosidase substrate that emits fluorescence only when it is cleaved by the enzyme to generate fluorophore 4-methylumbelliferone. Accordingly, we performed an assay on the *Mecp2^+/−^* RTT and CTRL MSC lysates to measure the 4-methylumbelliferone fluorophore production at an emission/excitation wavelength of 365/460 nm [73].

### 4.7. Apoptosis Detection

Apoptosis was detected with the fluorescein conjugated Annexin V Kit (Roche, Italy), following the manufacturer’s instructions. Apoptotic cells were identified with a fluorescence microscope (Leica Italia, Rome, Italy). To calculate the percentage of apoptosis in the *Mecp2^+/−^* and CTRL MSCs, we counted, in each experiment, a minimum of 1000 cells in different fields.

### 4.8. Necrosis Detection

Necrotic non-viable cells were detected by using Trypan blue staining. Briefly, we trypsinized the *Mecp2^+/−^* and CTRL MSCs and then we incubated them in a 0.2% Trypan blue solution for a few minutes. Non-viable cells (blue cells) were identified with an inverted microscope. To determine the percentage of dead cells, we counted, in each experiment, a minimum of 1000 cells in different fields.

### 4.9. RNA Extraction and RT-qPCR

Total RNA was extracted from the *Mecp2^+/−^* and CTRL MSCs using EuroGOLD Trifast (EuroClone S.p.A., Pero, Italy), following the manufacturer’s instructions. The mRNA levels of the interest genes were analyzed by quantitative reverse transcription polymerase chain reaction (RT-qPCR), as already reported [74]. Primer pairs for RT-qPCR were designed using Primer Express software (Thermo Fisher Scientific, Waltham, MA, USA). We used appropriate regions of HPRT cDNA as internal control [75]. Each RT-qPCR reaction was repeated at least three times. Primer sequences were as follows: *Hprt* (F) 5′-ACCTTCTATGAATGTTACTG-3′, (R) 5′-GATAAGCGACAATCTACC-3′; *Rb1* (F) 5′-CTTGAACCTGCTTGTCCTCTC-3′, (R) 5′-GGCTGCTTGTGTTCTCTGTATT-3′; *Rb2* (F) 5′-TCTCTTGGACGACGGAAG -3′, (R) 5′-CCTGGAACACTAACCTCACT-3′; *P16* (F) 5′-ATGTTGTTGAGGCTAGAGA-3′, (R) 5′-CCGTAGTTGAGCAGAAGA-3′; *P53* (F) 5′-CCTCATCCTCCTCCTTCC-3′, (R) 5′-CGACTGTGACTCCTCCAT-3′; *P21* (F) 5′-GGTTCCTTGCCACTTCTTAC-3′, (R) 5′-ACTGCTTCACTGTCATCCT-3′; *P27* (F) 5′-GGACTTGGAGAAGCACTG-3′, (R) 5′-TCCTGCCACTCGTATCTG-3′.

### 4.10. Treatment with Hydrogen Peroxide, Doxorubicin, and Ultraviolet Irradiation

Hydrogen peroxide (H_2_O_2_) treatment: *Mecp2^+/−^* and CTRL MSCs were treated for 1 h with 300 mM H_2_O_2_. Then, the medium was removed, and a complete culture medium was added. Finally, 48 h later, cells were collected for data analysis.

Doxorubicin treatment: *Mecp2^+/−^* and CTRL MSCs were treated with 1 μM doxorubicin in a complete culture medium for 24 h. Then, the medium was removed, and the cells were incubated for 48 h in a fresh medium before further analysis.

Ultraviolet (UV) irradiation: *Mecp2^+/−^* and CTRL MSC plates were irradiated with UV light by exposure to a germicidal lamp (peak sensitivity approximately 365 nm) in a tissue culture hood (15 mJ/cm^2^) for 7 min. Then, the medium was removed, and a complete culture medium was added. Finally, 48 h later, cells were collected for data analysis.

### 4.11. Immunocytochemistry for γ-H2AX Detection

The γ-H2AX (2577, Cell Signaling, MA, USA) was detected in the *Mecp2^+/−^* and CTRL MSC cultures according to the manufacturer’s instructions. Cell nuclei were stained with 4′,6-diamidino-2-phenylindole (DAPI) and then observed with a fluorescence microscope (Leica Italia, Italy). We calculated the percentage of γ-H2AX positive cells by counting a minimum of 500 cells in different microscope fields.

### 4.12. Workflow for Oxygen Consumption, ATP and Lactate Assays

1 × 10^6^ *Mecp2^+/−^* and CTRL MSCs were seeded in 150 mm cell culture dishes with α-MEM containing 10% fetal bovine serum, 2 ng/mL bFGF, 2 mM L-glutamine, 100 U/mL penicillin, and 100 μg/mL streptomycin (D0_growth medium). Two days after (D2_substrate-limited medium), the growth medium was replaced with the substrate-limited medium containing DMEM (A14430 Thermo Fisher Scientific, Waltham, MA, USA), 1% FBS, 0.5 mM glucose, 1mM GlutaMAX, and 0.5 mM carnitine. Following incubation for 24 h (T0_assay medium), the substrate-limited medium was replaced with the assay medium (PBS containing MgCl_2_ and CaCl_2_, 5 mM HEPES, 2.5 mM glucose, and 0.5 mM carnitine). After 30 min (T30_meteabolic inhibitor drug addiction) in a group of both Mecp2^+/−^ and CTRL MSCs, the metabolic pathway inhibitor drugs (48 mM 2d-glucose or 0.25 mM etomoxir or 3 μM BPTES) were added. Following 60 min (T90_substrate addition), the relative substrates (25 mM glucose or 200 μM palmitate or 4 mM glutamine) were added in all samples. After 60 min incubation (T150_assays), the oxygen consumption, ATP, and lactate assays were performed.

### 4.13. Oxygen Consumption Assay

We measured the intracellular oxygen levels in the *Mecp2^+/−^* and CTRL MSCs to evaluate carbohydrates, lipids, and glutamine contribution to oxygen consumption. The MitoXpress® Xtra assay (Agilent Technologies, Luxcel Biosciences, Cork, Ireland) allowed us to determine extracellular oxygen consumption rates (OCR). The assay is based on the use of an oxygen-sensing fluorophore that is quenched by O_2_ through molecular collision. Therefore, the amount of fluorescence signal is inversely proportional to the amount of extracellular O_2_ in the analyzed samples. We first determined basal respiration. Subsequently, we added oligomycin and trifluoromethoxy carbonylcyanide phenylhydrazone (FCCP), which target components of the electron transport chain (ETC) in the mitochondria to reveal key parameters of metabolic function. The addition of these components allowed us to determine oxygen employed for mitochondrial ATP production and maximal respiration, respectively. The OCR was determined according to the manufacturer’s guidance and instructions.

### 4.14. ATP Assay

We used the ATP Colorimetric/Fluorometric Assay Kit (BioVision, Milpitas, CA, USA) to measure ATP levels in the *Mecp2^+/−^* and CTRL MSC cultures supplemented with glucose, palmitate, or glutamine. The assay utilizes the glycerol phosphorylation to generate a product that is easily quantified by fluorometric (Ex/Em = 535/587 nm) methods. We performed measurements with the fluorometric assay in 96-well black bottom plates according to the manufacturer’s guidance and instructions.

### 4.15. Lactate Assay

Lactate production in the *Mecp2^+/−^* and CTRL MSCs was determined by the Lactate Assay Kit (Sigma-Aldrich Italy, Milan, Italy), an enzymatic assay that results in a fluorometric (λex = 535 nm/λem = 587 nm) product proportional to the lactate concentration. We performed measurements with the fluorometric assay in 96-well black bottom plates according to the manufacturer’s guidance and instructions.

### 4.16. Western Blotting

The *Mecp2^+/−^* and CTRL MSCs were lysed for 30 min at 4 °C using a buffer containing 0.1% Triton. Twenty μg of each lysate was processed by electrophoresis in a polyacrylamide gel and transferred onto a nitrocellulose membrane. The primary antibodies LC3 (ab51520, Abcam, Cambridge, UK) and GAPDH (G8795, Sigma Aldrich, Saint Louis, MO, USA) were used following the manufacturers’ instructions. Immunoreactive signals were detected using a horseradish peroxidase-conjugated secondary antibody (Santa Cruz Biotechnology, Dallas, TX, USA) and reacted with ECL plus reagent (GE Healthcare, Milan, Italy), a chemiluminescent substrate-based system.

### 4.17. Proteasome Activity

Proteasome activity in the *Mecp2^+/−^* and CTRL MSCs was evaluated by the 20S Proteasome Activity Assay Kit (Merck-Millipore Italy, Milan, Italy). It is a simple and convenient method that recognizes the substrate LLVY. The assay allowed us to detect fluorophore 7-Amino-4-methylcoumarin (AMC) after cleavage from the labeled substrate LLVY-AMC. The fluorescence from the free AMC can be measured using a fluorometer (380/460 nm). We performed measurements with the fluorometric assay in 96-well black bottom plates according to the manufacturer’s guidance and instructions.

### 4.18. Statistical Analysis

We evaluated statistical significance using the analysis of variance followed by the Student’s *t* and Bonferroni’s tests. For data with continuous outcomes, we used the mixed-model variance analysis and, in any case, we analyzed all data with the GraphPad Prism, version 5.01 (GraphPad, San Diego, CA, USA).

## 5. Conclusions

Our findings shed light on the dysfunctions related to impaired MECP2 activity that contribute to RTT symptoms. Unfortunately, no definitive treatments for RTT are available to date. We demonstrated that MECP2 deficiencies might lead to senescence and impaired mitochondrial energy production, which in turn may determine a decline in metabolism. Consistent with literature data, our results support the idea that mitochondrial energy production impairment could play an important role in the pathogenesis of RTT.

As a whole, our study suggests that setting up an anti-aging treatment in animal models of RTT may be the starting point to design an effective approach for patients. The treatments will be based on the use of mTOR inhibitors and/or molecules with anti-oxidant proprieties, such as natural bioactive compounds [76,77,78,79,80,81,82,83]. These treatments are under investigation for counteracting cellular senescence and hence slow organismal aging. The anti-senescence activities of these drugs could be potentially useful to delay or reduce RTT patients’ symptoms.

Moreover, a detailed study of biochemical pathways related to mitochondrial respiration could lead to new insights for coping with metabolic dysfunctions of RTT.

## Abbreviations

RTT	Rett syndrome
MECP2	Methyl-CpG binding Protein 2
MSCs	Mesenchymal Stromal Cells
WT	Wild Type
CKIs	Cyclin Kinase Inhibitors
DDR	DNA Damage Response
ATM	Ataxia Telangiectasia Mutated kinase
2-DG	2-deoxy-d-Glucose
BPTES	Bis-2-(5-Phenylacetamido-1,3,4-Thiadiazol-2-yl)Ethyl Sulfide
FCCP	Trifluoromethoxy Carbonylcyanide Phenylhydrazone
LDH	Lactate Dehydrogenase
LC3	Microtubule associated protein 1 Light Chain 3
TCA	Tricarboxylic Acid
α-MEM	Minimal Essential Media with alpha modifications
FBS	Fetal Bovine Serum
bFGF	basic Fibroblast Growth Factor
PBS	Phosphate Buffered Saline
4-MUG	4-Methylumbelliferyl-β-d-Galactopyranoside
RT-qPCR	quantitative Reverse Transcription Polymerase Chain Reaction
DAPI	4′,6-Diamidino-2-Phenylindole
OCR	Oxygen Consumption Rates
ETC	Electron Transport Chain
AMC	7-Amino-4-methylcoumarin
mTOR	mechanistic Target of Rapamycin
